# Biological functions of iduronic acid in chondroitin/dermatan sulfate

**DOI:** 10.1111/febs.12214

**Published:** 2013-03-28

**Authors:** Martin A Thelin, Barbara Bartolini, Jakob Axelsson, Renata Gustafsson, Emil Tykesson, Edgar Pera, Åke Oldberg, Marco Maccarana, Anders Malmstrom

**Affiliations:** Department of Experimental Medical Science, BMC, Lund UniversitySweden

**Keywords:** cancer, dermatan sulfate epimerase, DSE, DSEL, iduronic acid, inflammation, proteoglycans

## Abstract

The presence of iduronic acid in chondroitin/dermatan sulfate changes the properties of the polysaccharides because it generates a more flexible chain with increased binding potentials. Iduronic acid in chondroitin/dermatan sulfate influences multiple cellular properties, such as migration, proliferation, differentiation, angiogenesis and the regulation of cytokine/growth factor activities. Under pathological conditions such as wound healing, inflammation and cancer, iduronic acid has diverse regulatory functions. Iduronic acid is formed by two epimerases (i.e. dermatan sulfate epimerase 1 and 2) that have different tissue distribution and properties. The role of iduronic acid in chondroitin/dermatan sulfate is highlighted by the vast changes in connective tissue features in patients with a new type of Ehler–Danlos syndrome: adducted thumb-clubfoot syndrome. Future research aims to understand the roles of the two epimerases and their interplay with the sulfotransferases involved in chondroitin sulfate/dermatan sulfate biosynthesis. Furthermore, a better definition of chondroitin/dermatan sulfate functions using different knockout models is needed. In this review, we focus on the two enzymes responsible for iduronic acid formation, as well as the role of iduronic acid in health and disease.

## Introduction

**Fig. 1:**
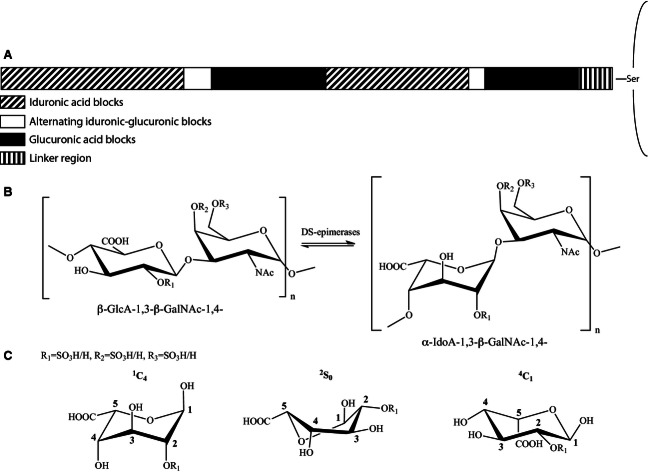
Structure of CS/DS and conformations of IdoA. (A) The domains of variable length containing blocks of IdoA, alternating IdoA and GlcA or blocks of GlcA. (B) The epimerase reaction. (C) Conformations of IdoA.

Dermatan sulfate (DS) is a glycosaminoglycan (GAG) that is distinguished from chondroitin sulfate (CS) by the presence of iduronic acid (IdoA), the C-5 epimer of d-glucuronic acid (GlcA). IdoA occurs in variable proportions in DS (Fig. 1A) and, as a result of the different position of the carboxyl moiety (Fig. 1B), it generates a more flexible polysaccharide chain, allowing specific interactions with several proteins and polysaccharides. To form CS/DS, three specific enzymes, dermatan sulfate epimerase 1 (DS-epi1), dermatan sulfate epimerase 2 (DS-epi2) and dermatan 4-*O*-sulfotransferse 1 (D4ST1), are required 1. These enzymes are differently organized in various tissues and, under different physiological conditions, they generate CS/DS of a very different structure. DS is found relatively late in the evolutionary tree and first appears in molluscs, sea urchins and sea cucumbers. It is then found in ascidians and in the whole vertebrate phyla 2. However, it is absent in *Caenorhabditis* *elegans* and *Drosophila* *melanogaster*. The present review presents the structure, function and biosynthesis of these structurally different CS/DS polymers and explains how they are modified in response to different physiological and pathological processes.

## Structure of CS/DS

CS/DS chains are found on at least 32 different core proteins forming proteoglycans (Table 1). Six of these are also substituted with heparan sulfate. Some of these proteoglycans, such as CD44, α5β1 integrin and collagen XV, are only part-time proteoglycans.

**Table 1:** CS/DS PGs and functions of the CS/DS chain. NA, not analyzed

PG	Presence of IdoA	Functions of PG	CS/DS binding proteins and CS/DS functions
*Extracellular matrix*			
Aggrecan	NA	Chondroskeletal morphogenesis, chondrocyte–matrix adhesion, cartilage hydration, neuronal cell aggregation 78	Water retention
Versican	IdoA+	Increases differentiation, motility, proliferation and metastasis 79,80. ECM assembly 81	FGF family, L- and P-selectin, chemokines
Decorin	IdoA+	TGF-β interaction 82, self-association 83, modulation of proliferation, survival, migration and angiogenesis 84, coagulation 60, LDL interaction 54, *Borrelia* invasion 65, α-defensin targeting 66, progeroid and Ehlers–Danlos syndromes 85	FGF2, FGF7, HGF, HCII, α2β1integrin, tenascin-X, fibril formation, DS:DS self-association 86
Biglycan	IdoA+	Interactions withTGF-β 87, BMP4/chordin 88, collagen I 89, associated with tumour in gastric tissue 90 and endothelial cells 91, involved in inflammation and development 92,93, neuronal survival 94, bone development and osteoporosis 95,96	HCII, FGF family
Epiphycan	IdoA+	Chondrocyte differentiation 97 and matrix organization in the growth plate 98	NA
Collagen IX	NA	Organization of cartilage 99, associated with fibroblasts in colon cancer	NA
Collagen XII	NA	Organization of cartilage and skin 100	NA
Collagen XIV	NA	Organization of cartilage and skin 101,102	NA
*Cell surface*			
Betaglycan	NA	TGF-β presentation 103,104 and suppression of cancer progression and metastasis 105, binds inhibin and suppresses activin signalling 106	NA
Syndecan-1	IdoA+	Regulation of tumour cell survival and proliferation, growth factor and cytokine binding, adhesion 107–109	Midkine, pleiotropin, FGF
Syndecan-3	NA	Role in human labour 110,111, adhesion, growth factors co-receptor, neurite outgrowth 112, expressed in tumour stromal vessels 113	NA
Syndecan-4	NA	Interaction with Frizzled7 and Dishevelled, regulates noncanonical Wnt signalling and convergent extension movements in *Xenopus* 114, regulates neural crest cells migration 115 and neural induction via extracellular signal-regulated kinase and protein kinase C pathways 116, adhesion, growth factors co-receptor 109, wound healing and angiogenesis 117, up-regulated in cancer and mediator of cell spreading 118	Midkine, pleiothropin, bFGF 109
CD44	IdoA+	Tumour growth, angiogenesis, metastasis, migration, HGF binding 119	Migration, HGF
NG2	NA	Regulates tumour cell growth, motility and survival 120	Differentiation, proliferation and motility, PDGF-AA and FGF2, adhesion 121
α5β1 integrin	NA	Fibronectin binding, regulation of adhesion and migration 122	NA
*Nervous system*			
Neuropilin-1	NA	Metastasis, neuronal guidance, regulation of cell migration 123	VEGF signalling
Neurocan	NA	Up-regulated in astrocytoma 124, neurite outgrowth, growth factors binding, brain ECM organization 125	N-CAM, HB-GAM, amphoterin
Phosphacan	IdoA+	Mediates migration and adhesion, differentiation of neuro stem cells 125–128	HB-GAM, amphoterin, midkine
Brevican	NA	Promotes glioma invasion 129,130, regulation of synaptic plasticity 131	Neuritogenic activity
Appican (AβPP isofom)	NA	Neuronal cell adhesion and migration, neurite outgrowth 132	Midkine, pleiotrophin
Neuroglycan C	NA	Cerebral development and neuritogenesis	NA
*Basal membranes*			
Perlecan	NA	Basal membrane stability, embryogenesis, cytokine interaction 133 interaction with FGFs, angiogenis 134	NA
Bamacan	NA	Basal membrane, regulator of angiogenesis 135, anchorage-independent growth 136	NA
Leprecan	NA	Kidney development, fibrillar collagen regulator 137	NA
Collagen XV	NA	Suppresses tumour growth 138	NA
*Intracellular*			
Serglycin	IdoA+	Inflammatory process 139	Cytokine binding and coagulation, granulocyte maturation
*Other proteoglycans*			
SRPX2	IdoA+	Overexpressed in gastrointestinal cancer, increases endothelial proliferation, cell signalling modulation, endothelial cell migration and angiogenesis 140	HGF
Endocan	IdoA+	Promotes tumour formation 141,142, mitogenic regulator, inflammation	HGF
Testican-1	NA	Inhibition of proteases, neurite extension 143	NA
Testican-2	NA	Promotes invasion and abrogates proteases inhibition of other proteins of the testican family 144	NA
Testican-3	NA	Inhibits invasion, regulates neurite development 145	NA
Bikunin	NA	Stabilization of ECM, activity in cumuli oophori, modulation of antiproteases 14,146	

CS is a long polysaccharide consisting of the repeating disaccharide units GlcA and *N*-acetyl-galactosamine (GalNAc), attached to serine residues of core proteins. The chains from eukaryotic organisms are extensively modified by sulfation, yielding six different disaccharides: GlcA-GalNac residues (O unit), GlcA-GalNAc-4-sulfate (A unit), GlcA-GalNAc-6-sulfate (C unit), GlcA-GalNAc-4,6-disulfated (E unit). The GlcA residue can also be sulfated at the 2-position giving rise to B units (GlcA-2-sulfated-GalNAc-4-sulfated) and D units (GlcA-2-sulfated-GalNAc-6-sulfated) 3. Even more complex sulfation patterns have been described in the invertebrate phyla 2.

An important modification is the epimerization of GlcA residues to IdoA residues by C-5 inversion at the polymer level of a (β-GlcA-1,3-β-GalNAc-1,4-)_n_ substrate (Fig. 1B) 4. Individual saccharide units in CS/DS can exist in different conformations depending on their structural arrangement. IdoA residues allow flexibility given their ability to switch between ^1^C_4_ (chair), ^2^S_0_ (skew boat) and ^4^C_1_ (chair) conformations (Fig. 1C), whereas GlcA residues are less flexible and exist in the ^4^C_1_ (chair) conformation 5. IdoA can occur in three different arrangements: (a) as a single IdoA-containing disaccharide surrounded by GlcA containing disaccharides; (b) in structures where they alternate with GlcA containing disaccharides or (c) in long blocks of adjacent IdoA-containing disaccharides (Fig. 1A). The sulfation pattern differs according to the IdoA distribution because IdoA blocks are mainly 4-sulfated with some adjacent sulfated IdoA residues (iB) close to the nonreducing terminal of the blocks 6,7. The short GlcA blocks are mostly 4-sulfated, whereas longer blocks also contain 6-sulfated GalNac residues 8. The resulting CS/DS chains therefore contain different domains that enrich their functional properties. The presence of alternating IdoA-GlcA or isolated IdoA has been overlooked in many cases. Furthermore, the content of IdoA varies within the same proteoglycan depending on the tissue of expression 6 and physiological conditions 9. This is the case for decorin, which is highly iduronated in skin. In bone decorin, however, IdoA is virtually absent 6. Given the fact that a chain containing IdoA always contains GlcA, the name CS/DS indicates the hybrid nature of the chain.

The structural characterization of CS/DS takes advantage of specific lyases such as chondroitinase ABC, AC and B, which specifically degrade galactosaminoglycans depending on the presence of IdoA or GlcA. The development of high-resolution HPLC systems with pre- or post-column fluorescent derivatization has enabled the separation and quantitation of the various building blocks 10,11. These methods can only determine the degree of sulfation and the occurrence of IdoA- and GlcA-blocks. However, detailed sequence analysis is not possible. The advent of sensitive MS with different fragmentation procedures has lead to promising results 12,13. Recently, the complete sequence determination of the chondroitin sulfate in bikunin has been accomplished 14.

## Biosynthesis of DS

DS-epi1 and DS-epi2 catalyze the formation of IdoA, the stereoisomeric form of GlcA, by repositioning the C5 carboxyl group in space (Fig. 1B). DS-epi1 (coded by the gene *DSE*) and DS-epi2 (coded by the gene *DSEL*) are both ubiquitously expressed and have common structural features 15,16.

**Fig. 2:**
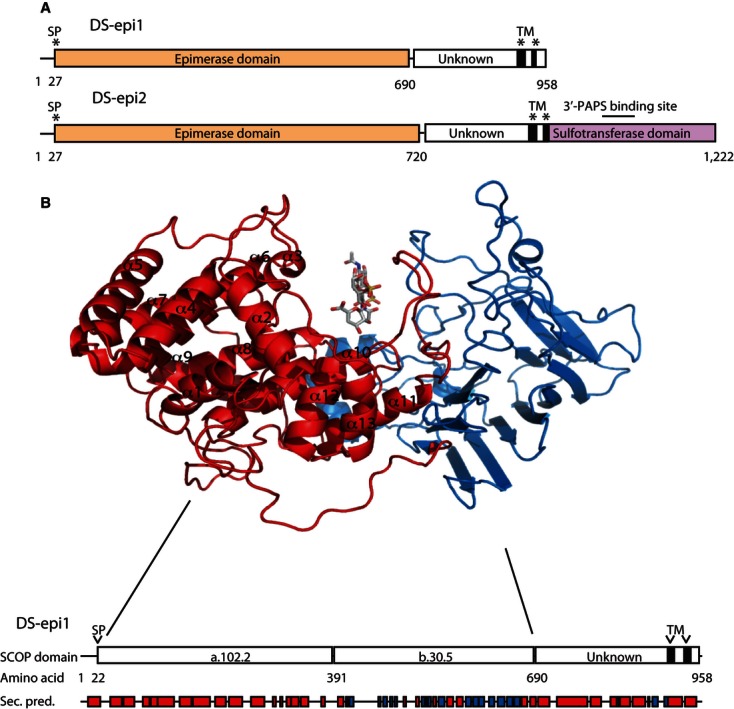
(A) DS-epi1 and DS-epi2 domain structures. (B) Three-dimensional modelling of the DS-epi1 epimerase domain based on the crystal structure of heparinase II. A chondroitin sulfate tetrasaccharide is positioned in the groove containing the active site.

DS-epi1 and 2 share a common N-terminal epimerase domain (Fig. 2A) with 51% amino acid sequence identity between the two enzymes. The secondary and tertiary structures of this domain in the two enzymes are very similar. DS-epi1 has a C-terminal domain of unknown function and three-dimensional structure. There is a similarly positioned domain in DS-epi2 with unknown function and structure. These two domains in the two epimerases do not have significant homology. In addition, in DS-epi2, there is a C-terminal domain, which has 16% amino acid identity with chondroitin-*O*-sulfotransferase 1, recognized in the database as a CS/DS–*O*-sulfotransferase domain (Fig. 2A), suggesting that DS-epi2 is an enzyme with dual epimerase and *O*-sulfotransferase activity. Other enzymes for GAG biosynthesis have been shown to accommodate dual activities 17,18. The functional epimerase domain of the DS epimerases comprises two structural domains: one mainly composed of α-helices and one of β-sheets (Fig. 2B). These two domains of DS-epi1 were modelled on the crystal structure of heparinase II 19. At their boundary, they form a groove, where the substrate is positioned. Some amino acids that are essential for enzyme activity have been identified and a catalytic mechanism has been proposed. Histidine 450 abstracts the C5 proton from one side of the sugar plane of GlcA. This is followed by cleavage or glycosidic linkage between GalNAc and GlcA to generate a C4–C5 double bond containing hexuronic acid intermediate. This structure is finally protonated by histidine 205 adding a hydrogen at the side of the sugar plane that is opposite to the abstraction side. Finally, the glycosidic link is recreated. As a result of the reaction, the carboxyl group has a different spacial orientation in the IdoA epimer than in the starting GlcA. A prerequisite for activity is the presence of at least three of the four N-glycans.

DSE and DSEL are on chromosomes 6 and 18, respectively 15,20. The exon/intron organization of the two enzymes is very different because DSE has six exons and the coding sequence spans five exons, whereas DSEL has only two exons (being the whole ORF present in the single exon 2).

The epimerase activity is highly expressed in the spleen, stomach, uterus, ovary, kidney and lung. In the brain, the activity is low and no activity is found in serum 21. By analyzing the total activity in tissues and mouse embryonic fibroblasts of DS-epi1^−/−^ and DS-epi2^−/−^ mice, it is possible to show that DS-epi1 is the predominant epimerase in most tissues, whereas DS-epi2 is the main epimerase in the brain 21,22. DS-epi2 also has a relatively high expression in the kidney.

**Fig. 3:**
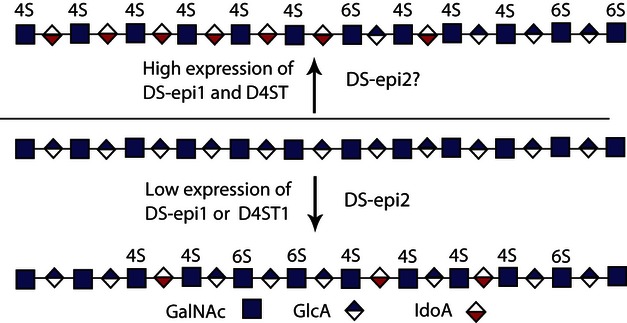
Formation of IdoA in CS/DS. The amount and distribution of IdoA depends upon the expression level of the DS epimerases and D4ST1.

The epimerase reaction is reversible, with an equilibrium of 9 : 1 (GlcA to IdoA) under *in vitro* conditions when the biosynthetic complex has been solubilized with detergent 4. On the other hand, CS/DS chains *in vivo* can contain a higher proportion of IdoA. This is assumed to be achieved through functional collaboration between DS-epi1 and D4ST1 (Fig. 3) 23. In support of this, transient down-regulation of D4ST1 results in a reduced IdoA content 24. Genetic mutations in D4ST1 found in a new type of Ehlers–Danlos syndrome (i.e. adducted thumb-clubfoot syndrome) also result in CS/DS of low IdoA content 25.

Little is known about the regulation of epimerase activity. Transforming growth factor (TGF)-β-stimulated fibroblasts have reduced levels of epimerase activity, a reduced expression of D4ST1 and an increased expression of C4ST1, resulting in CS/DS with a considerably lower amount of IdoA 26. This effect is further increased by combined treatment with TGF-β, epidermal growth factor and platelet-derived growth factor (PDGF) (9). In another study, PDGF promoted the migration of fibroblasts, comprising a mechanism that is proposed to involve the up-regulation of IdoA in the proteoglycan CD44 27.

The products of DS-epi1 and 2 are difficult to assess as a result of the complex interaction with D4ST1. DS-epi1 can generate long blocks of IdoA together with D4ST1 (Fig. 3). Down-regulation of D4ST1 resulted in the abrogation of IdoA-containing blocks without affecting overall epimerase activity 24. The role of DS-epi2 has been more difficult to assess. Overexpression of DS-epi2 increased IdoA in hybrid structures (Fig. 3). No increase of IdoA blocks was recorded upon overexpression of DS-epi2, whereas overexpression of DS-epi1 resulted in enhanced block formation 16. By contrast, down-regulation of DS-epi2 in fibroblasts decreased the proportion of IdoA blocks, although to a smaller degree than that obtained by down-regulation of DS-epi1. Data obtained from DS-epi1 knockout mice show that DS-epi2 mainly forms alternating structures 28. These data indicate that DS-epi2 might be primarily involved in the formation of isolated or alternating IdoA structures (Fig. 3).

Different proteoglycans produced by the same cell can vary greatly with respect to their IdoA content and distribution. For example, decorin and biglycan have been found to contain blocks of IdoA, whereas versican only has isolated IdoA. Other studies have suggested that the core protein regulates the activity of the DS epimerases. This was demonstrated by the generation of chimeric proteins of decorin, which has a high content of IdoA, and colony-stimulating factor, a part-time proteoglycan with a low content of IdoA. The chimeric decorin–colony-stimulating factor contained less IdoA than the unmodified decorin 29. This suggests that core proteins carry information that may direct the proteoglycan cores to compartments within the Golgi complex with different amounts of DS epimerase activity 30.

## Functions of IdoA as indicated by targeting of the two epimerases

**Fig. 4:**
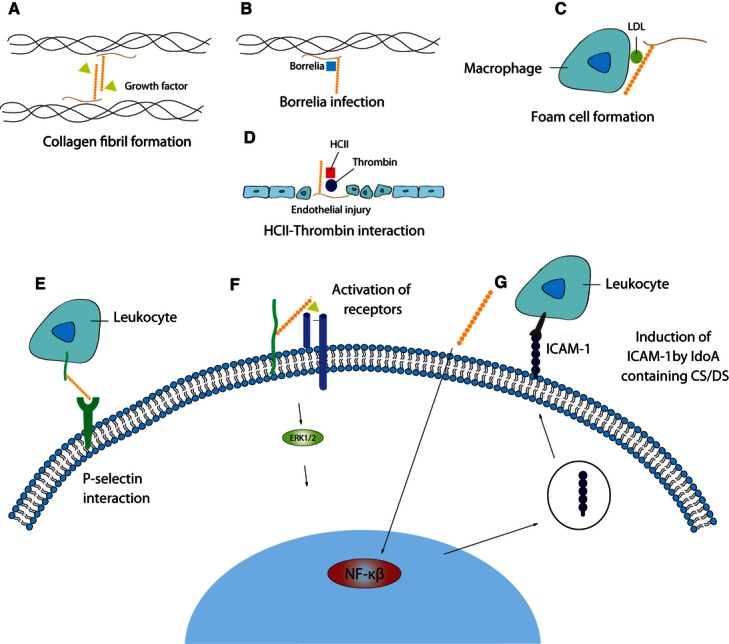
Overview of the functions of IdoA in CS/DS. Role of IdoA in the storage of cytokines growth factors and collagen fibril formation (A), *Borrelia* infection (B), atherosclerosis (C), coagulation (D), P-selectin-dependent leukocyte recruitment (E), activation of cytokine and growth factor receptors (F) and leukocyte recruitment by ICAM (G).

The phenotype observed in DS-epi1 knockout mice is dependent upon the genetic background. Using mice with a pure C57BL6 genetic background, all pups die perinatally, whereas, when using mice with a pure NFR background, approximately half of the pups die. The NFR pups have a retarded growth rate in the late embryological stages of development and, furthermore, ∼ 20% of the pups display gastroschisis, an abdominal wall-closure defect that presents intestines outside the body (R. Gustafsson, unpublished data). DS-epi1 depleted mice in a mixed 129Sv/C57BL6 genetic background have been investigated in more detail. The pups were born at a normal Mendelian frequency 28. At birth, they are smaller and have a crooked tail. Because decorin is a major proteoglycan involved in the organization of collagen fibrils in skin, this tissue was studied in more detail. DS-epi1^−/−^ skin was more fragile than the skin of wild-type mice. DS-epi1^−/−^ collagen fibrils were more heterogeneous in denaturation profiles and *in vitro* experiments showed that, in DS-epi1^−/−^ skin, decorin was the proteoglycan that was responsible for altered collagen structure (Fig. 4A). Electron microscopy showed that the diameter of DS-epi1^−/−^ fibrils was 85 nm compared to 62 nm for wild-type mice 28. In summary, iduronic acid in the CS/DS chain and particularly of IdoA blocks participates in skin collagen fibril maturation.

DS-epi2^−/−^ mice do not show any evident phenotype 22. The brain was analyzed because DS-epi2 is the predominant epimerase in this tissue 22,31. Accordingly, DS-epi2^−/−^ brains had a 90% reduction in epimerase activity. The brains of newborn mice contain little IdoA (2% of the total chain), and this was further reduced in DS-epi2^−/−^ mice. However, the brain extracellular matrix (ECM) architecture was unaltered. It would be interesting to determine whether more subtle phenotypes such as behavioural changes are present in DS-epi2^−/−^ mice.

Mice deficient in DS-epi1 and 2 were recently obtained in a mixed 129Sv/C57BL6 genetic background. A large proportion of the pups die perinatally, although a few survive until 7 weeks of age. Double knockout mice are dwarf and have approximately half the size and weight of their wild-type littermates.

Down-regulation of DS-epi1 has been achieved in the frog *Xenopus laevis* using morpholino injections (E. Pera, unpublished data). Several abnormalities were observed, such as the absence of the dorsal fin, which could be explained by the altered migration of neural crest cells into that anatomical structure.

## Genetic alterations affecting IdoA formation in humans

There are no mutations in DS-epi1 associated with human diseases. However, mutations in D4ST1, which functionally collaborates with DS-epi1 to make IdoA blocks (Fig. 3), result in adducted thumb-clubfoot syndrome 32. Mutations of D4ST1 result in reduced amount of IdoA in CS/DS 25, also resulting in a defect in collagen fibril maturation and reduced collagen strength 28. This autosomal recessive syndrome 33 is characterized by facial changes, contractures of thumbs and fingers, joint instability, skin hyperextensibility, and heart and kidney defects. Additionally, myopathy has been described in these patients 34.

DS-epi2 has been genetically associated with bipolar disorder, which is a disease affecting ∼ 1% of mankind 15. Interestingly, two single nucleotide polymorphisms predicted to change the amino acid sequence were present in the bipolar disorder group and not in the control group.

## The role of IdoA in stem cell development

Embryonic stem cells are obtained from embryos and can be maintained in cell cultures as pluripotent stem cell lines with a capacity to differentiate into whole embryos. Studies have shown a four- to six-fold increase of CS/DS during the differentiation of murine embryonic stem cells to embroid bodies and to extra embryonic endodermal cells. The formation of embroid bodies and extra embryonic endodermal cells was accompanied by a two- and four-fold increase of IdoA, respectively 35, suggesting a role for IdoA. The biosynthetic genes *DSE*, *DSEL* and *CHST14*, coding for D4ST1, were expressed at all stages. *CHST14* was also expressed in the extra embryonic endodermal cells. However, the detailed structure of CS/DS, as well as its functions, still needs to be determined.

CS/DS is enriched in the neural stem cell niche and has been shown to play important role in the differentiation of neural progenitor cells 36. Its importance has been demonstrated in progenitor cells from mice with ablations of C4ST1 (a 4-*O*-sulfotransferase acting on GlcA-containing sequences) and D4ST1. Down-regulation of D4ST1 resulted in the abrogation of IdoA blocks, as well as decreased neurogenesis and proliferation and a change in the expression of cell surface receptors for fibroblast growth factor (FGF)-2 and epidermal growth factor, whereas C4ST1 deficiency did not affect these processes 37. The importance of IdoA motifs was further underlined by the fact that mRNA expression of the DS epimerases was higher in differentiated neurones than in precursor stem cells 38.

## IdoA-containing structures in brain development

CS/DS structures are implicated in brain development 39 and injury to the central nervous system 40. During development, IdoA-containing structures (iA, iB, iE and iD) are ubiquitous in different parts of the brain 31,41, although at low concentrations. Indeed, CS/DS brains of newborn mice comprise only 2% iduronic acid 22. The CS/DS bioenzymatic machinery is carefully regulated during brain development, resulting in a large variation of IdoA-containing structures. For example, in the cerebellum, iD decreases and iB increases from newborn to adult age 31. Interestingly, the embryo-derived CS/DS shows a greater binding of FGFs (FGF-2, -10 and -18), pleiotrophin, midkine, vascular endothelial growth factor (VEGF) and hepatocyte growth factor (HGF) than CS/DS from the brains of adult animals 42.

## The role of IdoA in CS/DS under pathological conditions

### Inflammation

The involvement of CS/DS in inflammation has been extensively explored, whereas the role of IdoA is not well defined 43,44. The inflammatory response initiated by infection or injury results in diverse processes, involving cell recruitment, extravasation and cell/pathogen clearance. For example, during wound healing, CS/DS is reported to be the dominating GAG in wound fluid 45,46. FGF2 and FGF7 are two important growth factors during wound repair and they have been shown to preferentially bind to IdoA-containing motifs in CS/DS, promoting proliferative processes (Fig. 4A,F). CS/DS has been proposed, in combination with FGF-10, as a pharmacological accelerator of wound closure as a result of its capacity to stimulate re-epithelialization 47. CS/DS can potentially affect several steps during cell recruitment. For example, CS/DS has been shown to interact with P-selectin, which is expressed on endothelial cells and platelets 48 (Fig. 4E). CS/DS is reported to influence the recruitment of polymorphonuclear cells in a thioglycollate-induced inflammatory model in a supposedly P-selectin manner 49. RANTES, a leukocyte-recruiting chemokine, also interacts with IdoA-containing segments in CS/DS 50. An essential step during extravasation is the increased expression of intercellular adhesion molecule-1 (ICAM-1) on endothelial cells. IdoA in CS/DS induces endothelial expression of ICAM-1 mediated by nuclear factor-kβ 45 (Fig. 4G). Interestingly, macrophages are reported to produce CS/DS containing up to 70% of IdoA 51. After lipopolysaccharide stimulation, macrophages predominantly secrete CS/DS, either as free chains or bound to the serglycin core protein.

### Immune response

Autoimmunity is a result of a disarray in the immune response, which becomes directed towards its own tissue and cells. B cells participate in autoimmunity by the production of antibodies and presentation of self-antigens to T cells. IdoA motifs in CS/DS are reported to augment the proliferation of B1-a cells and increase their autoantibody production 52. IdoA in CS/DS interacts with components from apoptotic and dead cells and forms complexes that enhance autoantibody production. IdoA-containing structures in CS/DS bind autoantigens, which were enriched after CS/DS-affinity chromatography of cellular lysates. Two hundred autoantigens were identified by MS and could be used in western blot experiments to detect different autoantibody patterns of diagnostic value in patient sera 52,53. Further studies are needed to clarify the physiological role of CS/DS in the generation of natural autoantibodies.

### Atherosclerosis

Atherosclerosis, an inflammatory-driven disease, is characterized by the accumulation of cholesterol in arterial blood vessels, resulting in thicker and more fragile artery vessels. Binding of low-density lipoprotein (LDL) to GAGs is considered to be one of the steps in the onset of this disease 54. The GAG interaction enhances LDL uptake by macrophages, leading to the formation of foam cells (Fig. 4C). IdoA both in CS/DS and heparan sulfate is reported to enhance the binding of VLDL and LDL 55,56. Recently, it was reported that an antibody against CS/DS inhibited the LDL–CS/DS interaction and inhibited LDL oxidation *in vitro* 57. Furthermore, the injection of anti-CS/DS antibody in an atherosclerosis model of ApoE^−/−^ mice resulted in decreased arteriosclerotic lesions 58.

### Coagulation

Coagulation is essential under normal physiological conditions and several pathological conditions (e.g. cancer, atherosclerosis and sepsis) have enhanced coagulation. Thrombin, a serine protease, catalyzes the conversion of fibrinogen to fibrin, which forms blood clots in conjunction with platelets. Heparin cofactor II (HCII) is a thrombin inhibitor and the only known serpin to be activated by IdoA-containing CS/DS (Fig. 4D). The HCII binding site to CS/DS differs from that to HS 59. The HCII binding structures in CS/DS contain IdoA-2S-GalNAc-4S 60 or GlcA-GalNAc-4,6-disulfated 61 in hexa- and octasaccharides as minimal binding motifs. The complex CS/DS-HCII is considered to be the major anticoagulant system after injury of the vessel wall 60,62,63. CS/DS containing 2-*O*-sulfated IdoA also controls coagulation by activating protein C 64.

### Infection

CS/DS is involved in bacterial infections. *Borrelia* (causing Lyme disease) was shown to use the core protein of decorin, as well as its CS/DS side chain, as a binding target in the initial phase of infection 65 (Fig. 4B). CS/DS released from decorin by proteases produced by *Pseudomonas*, *Enterococcus* and *Streptococcus* 66 targets α-defensin and inhibits its bactericidal activity. The optimal structure for interaction to α-defensin is a motif containing a mix of IdoA and GlcA, which is found in decorin present in fibrous connective tissue 66.

## IdoA motifs in cancer

CS/DS is implicated in several cancer-promoting processes, such as cell proliferation and metastasis 3. DS-epi1, previously named SART2 (squamous cell carcinoma antigen recognized by T cell 2), is highly expressed in many tumours and cell lines 20. DS-epi1 expressed by cancer cells was recognized by HLA-A24-restricted and tumour-specific cytotoxic lymphocytes. Peptides from DS-epi1 were used in peptide-based immunotherapy phase I clinical trials for prostate cancer 67, glioblastoma multiforme 68 and hepatocellular carcinoma 69 with moderate success. We have established that DS-epi1 is not tumour specific because DS-epi1 is ubiquitously expressed in normal tissues 21. Squamous cell carcinoma from oesophagus contains epimerase activity that is increased four- to five-fold compared to normal oesophagus 13. DS-epi1 is localized both in stroma surrounding the tumour and in cancer cells. To investigate the role of IdoA, DS-epi1 was stably down-regulated in oesophagus squamous carcinoma cell lines using shRNA sequences. IdoA was shown to facilitate the binding of HGF to its receptor and was essential for cMET-dependent signalling 13 (Fig. 4F). In addition, DS-epi1 down-regulated cells displayed fewer cytoplasmic stress fibres than control cells. Furthermore, the focal adhesion complexes were evenly distributed at the cell surface in DS-epi1 down-regulated cells compared to control cells, which displayed focal adhesion complexes predominantly at the leading edge. This resulted in less migration and invasion of DS-epi1 down-regulated cells compared to control cells 13.

Different CS/DS structures mediate diverse function during cancer development. The sulfation pattern of CS/DS in cancer differs from normal tissue. For example, 6-*O*-mono-sulfated disaccharides are accumulated in tumours compared to normal tissues, whereas 4-*O*-mono-sulfated disaccharides are reduced 70. During metastasis, CS/DS disaccharides sulfated at positions 4 and 6 (E units) present on the surface of cancer cells facilitate colonization of the lung and liver 71,72. The process might be mediated by the receptor RAGE, which is highly expressed in the lung 73. Another pro-metastatic activity of the E units on cancer cells could be a result of the capability to bind platelet P-selectin 49, resulting in the formation of tumour microemboli. These cell–platelet aggregates protect cancer cells against elimination by the immune system. IdoA in CS/DS is also known to mediate P-selectin binding. Two CS/DS structures containing IdoA (iB units or iD units), as isolated from marine animals, inhibit metastasis in a P-selectin-dependent manner in a metastatic tumour model 49. Several studies report that CS/DS structures mediate growth factor and chemokine binding. IdoA is essential for HGF-mediated binding and an IdoA-containing tetrasaccharide is the minimum structure required to confer affinity 74. Exogenously added IdoA-containing motifs inhibit the proliferation of normal and malignant cells 75. Elimination of CS/DS on the cancer cell membrane by chondroitinase B inhibits the migration and invasion of tumour cells 76.

## Future perspectives in research and clinical therapy

Still largely unknown is how the complex structure of CS/DS is formed and how it is regulated. A key question is the organization of the biosynthetic enzymes in the Golgi and how this organization is modulated in different cells and tissues. The role of the two different epimerases, DS-epi1 and 2, as well as that of D4ST1, needs to be clarified.

Different functions of IdoA have been found both *in vitro* and *in vivo*. The human situations where DS-epi1 expression is changed in tumours and where D4ST1 mutations lead to deranged connective tissue have been highlighted. The importance of IdoA is evident from observations of DS-epi1 KO mice, which die perinatally and/or present gastroschisis. Furthermore, a decrease of IdoA leads to an altered collagen structure, resulting in a decreased tensile strength. Provocation of mice with targeted DS-epi1 and 2 will most likely provide more information about other biological functions of IdoA. Other data indicate the importance of IdoA in cytokine activity and storage, cell proliferation and migration, the control of coagulation, the formation of autoantibodies, the control of stem cell stability and differentiation.

In disease, IdoA contributes to cancer progression and infection. New avenues for future therapies have been tested, such as vaccination against cancer 67–69, or are warranted to control infection 65,66 and cancer 13,76,77. DS epimerases inhibitors could be used in cancer and fibrosis, as well as to guide stem cell differentiation 3.

## Abbreviations

CS: chondroitin sulfate
D4ST1: dermatan sulfate 4-*O*-sulfotransferase 1
DS: dermatan sulfate
DS-epi1: dermatan sulfate epimerase 1
DS-epi2: dermatan sulfate epimerase 2
ECM: extracellular matrix
FGF: fibroblast growth factor
GAG: glycosaminoglycan
GalNAc: *N*-acetyl-galactosamine
GlcA: d-glucuronic acid
HCII: heparin cofactor II
HGF: hepatocyte growth factor
IdoA: iduronic acid
INF-γ: interferon-γ
PDGF: platelet-derived growth factor
TGF: transforming growth factor
VEGF: vascular endothelial growth factor
